# Patterns of Statin Prescribing

**DOI:** 10.1371/journal.pmed.0020149

**Published:** 2005-05-31

Coronary heart disease (CHD) is the leading cause of morbidity and mortality in developed countries, and identifying and treating patients with high cholesterol has an essential role in the prevention of CHD. Therapeutic lifestyle changes are important for general reduction of risk overall, but patients who are more likely to develop CHD, or who have high cholesterol, should be treated with statins. Statins inhibit HMG-CoA reductase—a key enzyme in the cholesterol synthesis pathway.

Although we know that not all patients who may benefit from statins receive treatment, there is limited information on how patients are treated according to their estimated risk of developing CHD. Patients may be classified as at low, medium, or high risk of developing CHD according to the presence of CHD, some other medical conditions (for example, diabetes), and major risk factors including cholesterol level, smoking, lifestyle, and family history. Jun Ma and colleagues analyzed data from the National Ambulatory Medical Care Survey and the outpatient department component of the National Hospital Ambulatory Medical Care Survey to identify changes in treatment from 1993 to 2002, and to identify current clinical practice. These surveys have been validated against other data sources, and used in past research on cholesterol management.

The researchers found that between 1993 and 2002 the use of statins increased nearly 5-fold, from 9% to 49%, in ambulatory visits by patients with high cholesterol, but then declined to 36% in 2002. Overall, the use of statins was three times more likely in 2001 and 2002 than in 1995 and 1996. Patients at high risk of CHD were more likely to use statins than other patients. However, among patients whose visit had a reported high cholesterol, only 50% of patient visits at high risk of CHD and 44% of those at moderate risk were prescribed statins in 2002, well below the recommendations of current guidelines. Less than half the patient visits that arguably represent optimal opportunities for counseling services received counseling about how they might change their lifestyle. The study also showed inequities in use of statins for patients with different social and clinical characteristics, with lower usage in younger patients, females, African-Americans, and patients cared for by doctors who are not cardiologists.

As the authors declare, the study was funded by a maker of one of the statins, but the information acquired is of general interest. Persistent gaps in statin therapy suggest a need for improved identification of patients who may develop CHD, and treatment with statins when indicated, the authors say. A particular focus should be patients who are at risk of developing CHD. Education should be aimed at improving the practice of physicians—above all, those who are not heart specialists—so that they adhere to evidence-based medicine and published guidelines for cardiovascular risk reduction. In an accompanying Perspective (DOI: 10.1371/journal.pmed.0020131), Fiona Turnbull from the George Institute for International Health says that physicians need to move away from making treatment decisions based on single risk factors and instead use an approach based on absolute risk. “An understanding of the concept of ‘absolute risk’—the probability of a patient developing a cardiovascular event over a specified time period—is crucial,” she says.

